# Silent Synapse-Based Mechanisms of Critical Period Plasticity

**DOI:** 10.3389/fncel.2020.00213

**Published:** 2020-07-17

**Authors:** Weifeng Xu, Siegrid Löwel, Oliver M. Schlüter

**Affiliations:** ^1^Department of Neuroscience, Brown University, Providence, RI, United States; ^2^Carney Institute for Brain Science, Brown University, Providence, RI, United States; ^3^Department of Systems Neuroscience, Johann-Friedrich-Blumenbach Institute for Zoology & Anthropology, University of Göttingen, Göttingen, Germany; ^4^Campus Institute for Dynamics of Biological Networks, University of Göttingen, Göttingen, Germany; ^5^Collaborative Research Center 889, University of Göttingen, Göttingen, Germany; ^6^Department of Neuroscience, University of Pittsburgh, Pittsburgh, PA, United States; ^7^Department of Psychiatry and Psychotherapy, University Medical Center Göttingen, Göttingen, Germany

**Keywords:** environmental enrichment, dark exposure, spine elimination, monocular deprivation, unsilencing, refinement, innate synapse, gestalt synapse

## Abstract

Critical periods are postnatal, restricted time windows of heightened plasticity in cortical neural networks, during which experience refines principal neuron wiring configurations. Here, we propose a model with two distinct types of synapses, ***innate synapses*** that establish rudimentary networks with innate function, and ***gestalt synapses*** that govern the experience-dependent refinement process. Nascent gestalt synapses are constantly formed as AMPA receptor-silent synapses which are the substrates for critical period plasticity. Experience drives the unsilencing and stabilization of gestalt synapses, as well as synapse pruning. This maturation process changes synapse patterning and consequently the functional architecture of cortical excitatory networks. Ocular dominance plasticity (ODP) in the primary visual cortex (V1) is an established experimental model for cortical plasticity. While converging evidence indicates that the start of the critical period for ODP is marked by the maturation of local inhibitory circuits, recent results support our model that critical periods end through the progressive maturation of gestalt synapses. The cooperative yet opposing function of two postsynaptic signaling scaffolds of excitatory synapses, PSD-93 and PSD-95, governs the maturation of gestalt synapses. Without those proteins, networks do not progress far beyond their innate functionality, resulting in rather impaired perception. While cortical networks remain malleable throughout life, the cellular mechanisms and the scope of critical period and adult plasticity differ. Critical period ODP is initiated with the depression of deprived eye responses in V1, whereas adult ODP is characterized by an initial increase in non-deprived eye responses. Our model proposes the gestalt synapse-based mechanism for critical period ODP, and also predicts a different mechanism for adult ODP based on the sparsity of nascent gestalt synapses at that age. Under our model, early life experience shapes the boundaries (the gestalt) for network function, both for its optimal performance as well as for its pathological state. Thus, reintroducing nascent gestalt synapses as plasticity substrates into adults may improve the network gestalt to facilitate functional recovery.

## Introduction

Children learn differently than adults. They learn faster, and the skills that they acquire, stay with them for the rest of their lives. For example, children can achieve native-like proficiency in their second language if it is learned at young ages, but such ability is generally diminished after puberty. The learned second language in adults generally cannot shake off the accent. Thus, acquiring a language as a native depends on the age it is learned and less on the years experiencing it (Birdsong, 2018). The learning process in children is ultimately the acquisition and storage of information in the neural network for future reference, extraction, and modification. During so-called critical periods of restricted time windows in postnatal development, experience refines neural networks to set their foundation to optimally perform their dedicated function after continuous training later on. This process has been demonstrated across model systems and modalities. For example, songbirds learn to sing from early auditory experiences during critical periods, and if they do not have the chance to listen to a tutor of their species during that restricted time window, they will only acquire a rudimentary song (Brainard and Doupe, 2002; Prather et al., 2017). Experience can, however, also be maladaptive, e.g., if vision is compromised by an opaque lens, or if subjects are exposed to improper language in social contexts, early life experiences may lead to long-lasting impairments of vision or language skills, respectively (Grimshaw et al., 1998; Webber and Wood, 2005; Birch, 2013; Feldman et al., 2019).

In mammalian brains, the formation of cortical neural networks is initially guided by genetic programs, molecular pathfinding, and spontaneous neuronal activity patterns to establish innate functionality. These early networks are immature and rudimental and need a further experience-dependent specification of the excitatory network architecture for instructive functionality (Changeux and Danchin, 1976; Singer, 1995; O’Leary et al., 2007; Zipursky and Sanes, 2010). Experience-dependent cortical plasticity is broadly defined as the ability of cortical networks to encode information by long-lasting changes of their neural representations, and thus to adapt to ever-changing environments. The concept of critical period plasticity was pioneered by Hubel and Wiesel, who described experience-dependent plasticity of the functional architecture in the cat and rhesus monkey primary visual cortex (V1) (Hubel and Wiesel, 1963; Wiesel and Hubel, 1963a, b; LeVay et al., 1980). While zebra finch song learning (Aamodt et al., 1996; Roberts et al., 2010) and barn owl auditory localization (Feldman et al., 1996; Feldman and Knudsen, 1997) are also compelling experimental models, further molecular mechanistic driven studies are sparse due to technical limitations.

In the mammalian visual system, neurons in the binocular zone of V1 respond to visual inputs from both eyes (Mrsic-Flogel et al., 2007). Depriving one eye of vision by suturing it shut for a few days during postnatal development (monocular deprivation, MD), shifts the ocular dominance of binocular V1 neurons toward the non-deprived (experienced, also referred to as open) eye. This phenomenon is termed ocular dominance plasticity (ODP) and is the *in vivo* reference experimental model for studying cortical plasticity. ODP describes a network phenomenon that transcends from cats (Wiesel and Hubel, 1963b; Shatz and Stryker, 1978; Jones et al., 1984), monkeys (Hubel et al., 1977; Blakemore et al., 1978), and ferrets (Issa et al., 1999), to rodents such as rats (Rothblat et al., 1978) and mice (Dräger, 1978). ODP exploits the fact that visual experience can be reliably manipulated and leads to measurable modifications in V1-networks.

The visual impairments induced by MD before and throughout the critical period, mirror amblyopia in patients with impaired vision in one eye during their childhood (e.g., a cataract) (Prusky and Douglas, 2003; Webber and Wood, 2005; Birch, 2013). While the degree of recovery of visual features in animal species differs, it is generally accepted that full functional recovery is not achieved if binocular vision is only restored in adulthood. Therefore, understanding the cellular and molecular mechanisms underlying critical period plasticity is of utmost importance for developing causal therapeutic approaches to correct early network refinement defects. Furthermore, it is conceivable that those approaches might prove to be efficient also for inducing functional recovery in other neurodevelopmental disorders or after brain lesions caused by stroke or trauma.

**Box 1. Approaches to measure ocular dominance plasticity.**

To analyze ODP, visual responses are assessed using various techniques that report different parameters of network activity. These techniques include single-unit recordings (Dräger, 1978; Bear and Singer, 1986; Gordon and Stryker, 1996; Hensch et al., 1998; Fagiolini and Hensch, 2000), intrinsic signal optical imaging (Kalatsky and Stryker, 2003; Cang et al., 2005) visually evoked potentials (VEPs) (Sawtell et al., 2003; Frenkel and Bear, 2004) and two-photon calcium imaging (Mrsic-Flogel et al., 2007; Kameyama et al., 2010; Kuhlman et al., 2013).

Single unit recordings assess the proportion of the recorded cells, responding preferentially to the deprived eye versus the non-deprived eye, and the contralateral bias index (CBI) or ocular dominance index (ODI) is used to quantify ocular dominance shifts by relating the difference in evoked spike numbers. This analysis measures the output, i.e., action potentials from the recorded V1-cells, and reports the proportion of the population preferring one eye over the other. The resulting ocular dominance shift towards the non-deprived eye after MD can be due to either a reduction of the deprived eye responses, or to an increase of the non-deprived eye responses, or to both. In an attempt to quantify changes specific to either eye, responses have been normalized to the firing rate (Bukhari et al., 2015) which is based upon the assumption that the baseline firing rate does not change between control and MD animals. However, single unit recordings typically report a relative change in the firing rate between non-deprived and deprived eye as the CBI.

Two-photon calcium imaging reports changes in calcium indicator fluorescence following action potentials as a proxy for neuronal activity. Similar to single unit recordings, this approach can report whether a neuron prefers one eye over the other, and the CBI is typically calculated. Additionally, the amplitude of the fluorescence change is used as a proxy to compare neuronal responses to either eye’s input in binocular V1 of control and MD animals after normalizing to baseline fluorescence intensity (Mrsic-Flogel et al., 2007; Kameyama et al., 2010; Kuhlman et al., 2013). In contrast to single unit recordings, calcium imaging samples a more unbiased population of neurons, whereas single unit recordings typically sample the most active neurons. Calcium imaging is typically limited to superficial layers of the cortex, and targets neurons in layers 2/3 of V1 (Mrsic-Flogel et al., 2007; Kameyama et al., 2010; Kuhlman et al., 2013). In contrast, single unit recordings allow measurements in all cortical layers, but results are often pooled across layers. It should be noted that the intracellular calcium rise in the neurons is an indirect consequence of action potential-driven membrane depolarization, thus is inherently delayed from the neuronal spike and often lacks single action potential resolution. Additionally, the calcium indicators and the fluorescence detection approaches also introduce further temporal delay of the signal measured versus the immediate neuronal responses. These technical differences need to be taken into consideration when interpreting MD-induced circuit plasticity as mechanistic differences between layers are apparent (Trachtenberg et al., 2000; Rao and Daw, 2004; Crozier et al., 2007; Liu et al., 2008; Fong et al., 2020).

VEPs are complex extracellular field potential recordings induced by visual stimulation. Using one-dimensional current source density analysis to track the signal along the cortical depth, the VEPs induced by visual stimulation are primarily driven through thalamocortical synaptic inputs to layer 4 neurons (Sawtell et al., 2003), but also include population spikes and local network activities in the later phase of the VEP waveform. Chronic VEP recordings have been used to longitudinally track the collective activities of the recorded cell population in rodents (Sawtell et al., 2003; Frenkel and Bear, 2004).

Intrinsic signal optical imaging exploits the fact that active neurons consume more oxygen than inactive ones, thereby locally increasing the concentration of deoxyhemoglobin, which absorbs more of the red light that is shone onto the cortical surface for monitoring the visually stimulated activity changes in mostly superficial V1 (Kalatsky and Stryker, 2003; Cang et al., 2005). Absolute values of intrinsic signals are tracked in individual mice before and after MD through chronic imaging (Kaneko et al., 2008b; Greifzu et al., 2014) or they allow quantitative eye-specific comparisons between control and experimental animals (Cang et al., 2005). Both VEP recordings and intrinsic signal optical imaging measure population responses induced by visual stimulations of either eye. Both methods are used to monitor the specific changes in responses to visual stimulation of the deprived or the non-deprived eye, i.e., whether MD induces either deprived eye depression or non-deprived eye potentiation, or both to cause ODP. However, both approaches lack resolution to monitor individual neuronal responses. Furthermore, given the complexity of the signals, when analyzing the population responses, using VEP recordings and intrinsic signal optical imaging, the underlying cellular and circuit mechanisms for the same measured responses may be different.

In particular, by investigating mouse models using an array of experimental approaches (Box 1), the field has gained mechanistic insights into the molecular and cellular mechanisms of ODP, summarized in Table 1. Emerging evidence implicates that cortical plasticity, such as ODP, is regulated and controlled by distinct mechanisms during the critical period, as opposed to that in adult (Hooks and Chen, 2007; Espinosa and Stryker, 2012; Levelt and Hübener, 2012; Hensch and Quinlan, 2018; Stryker and Löwel, 2018). Here, we specify the key features for critical period plasticity in the binocular region of mouse V1, outlining a new model with dedicated synapse categories and states: ***innate*** versus ***gestalt synapses***, and silent, unsilenced, versus stabilized state. We will first outline our model for the refinement of excitatory neural networks during critical periods, and then elaborate on how the model is supported by our recent studies and the current body of literature. We will then describe the implications for the cellular mechanisms of ODP and perception. While our discussion is focused on ODP as an experimental model for cortical plasticity and critical periods, we also survey the different expression mechanisms of cortical plasticity at different developmental stages and address results that highlight the general applicability of the discussed principles.

**TABLE 1:** Ocular dominance plasticity in mice.

Age^#^	Genotype/manipulation	Treatment^∗^	Outcome			Reference and method
			CBI^*a*^ or ODI^*b*^	response to		
				deprived eye	non-deprived eye	
P11–12	WT	P11-12 until 6 weeks / one year	Decrease	-	-	Dräger, 1978^1^
P19, 23, 28, 32	WT	4 days	Decrease	-	-	Gordon and Stryker, 1996^ 1^
P36			n.s.	-	-	
centered P28		5-10, >14 days	Decrease	-	-	
P23	WT	5 days	Decrease	-	-	Hensch et al., 1998^ 1^
	GAD65 KO		n.s.	-	-	
P16, 23	WT	4 days	Decrease	-	-	Fagiolini and Hensch, 2000^1^, also see Iwai et al., 2003^ 1^
P46			n.s.	-	-	
P16, 23, 46	GAD65 KO		n.s.	-	-	
	GAD65 KO, Diazepam^*c*^		Decrease	-	-	
P16	WT	14 days	Decrease	-	-	
P46, 120			n.s.	-	-	
P16, 46, 120	GAD65 KO		Decrease	-	-	
P16	WT Diazepam^*c*^	4 days	Decrease	-	-	
P46			n.s.	-	-	
P45	GAD65 KO, Diazepam^*d*^	15 days	n.s.	-	-	
P21	WT	3 days	Decrease	Decrease	n.s.	Sawtell et al., 2003^2^
P38			n.s.	-	-	
P21, 28, 38, 45-52, 60-90		5 days	Decrease	-	-	
P72–90			Decrease	n.s.	Increase	
adult		up to 15 days	Decrease	-	Increase	
	conditional GluN1 KO (G35-3 CRE line x NR1 flx/-)	up to 15 days	n.s.	-	n.s.	
P28	WT	5–7 days	Decrease	Decrease	Increase	Frenkel and Bear, 2004^21^
P45, P120	NgR -/-, NogoA/B -/-	4 days	Decrease	-	-	McGee et al., 2005^ 1^
P26–28	WT	2, 4, 6–7 days	Decrease with >4 days MD	-	-	Cang et al., 2005^3^
P23–27 (CP)	WT	4 days	Decrease	Decrease	Decrease	Hofer et al., 2006^ 1,3^
P60–120 (adult)	WT with 4–5 days CP MD	3 days	Decrease	n.s.	Increase	
P27–28	WT	1 day	-	n.s.	n.s.	Mrsic-Flogel et al., 2007^4^
		2–3 days	-	Decrease in binocularly responding neurons andincrease in deprived eye responding neurons	n.s.	
		4–7 days	-		Increase	
P16–18 (pre CP)	WT	4–5 days	n.s.	n.s.	n.s.	Sato and Stryker, 2008^3^
P24-29 (CP)			Decrease	Decrease	n.s.	
	WT, CPP		n.s.	n.s.	n.s.	
12–13 week (post CP)	WT		n.s.	n.s.	n.s.	
starting at P24-29 (CP)	WT	7–22 days	Decrease	Decrease	Increase	
12–13 week (post CP)			Decrease	n.s.	Increase	
	WT, CPP		n.s.	n.s.	n.s.	
P26–27	TNFα^−/−^	5 days	Decrease	Decrease	n.s.	Kaneko et al., 2008b^3^
P25	WT	4 days	Decrease	-	-	Lehmann and Löwel, 2008^3^
P95		4 days	n.s.	-	-	
		7 days	Decrease	-	-	
P130, P215		4, 7 days	n.s.	-	-	
P27–28	WT	2 days	Decrease in ENs and INs	Decrease in ENs	Increase in ENs and INs	Kameyama et al., 2010^4^
P57–62		7 days	Decrease in ENs and INs, more in INs	Decrease in INs	Increase in ENs	
P26–27 (CP)	WT	3 days	Decrease	Decrease	n.s.	Ranson et al., 2012^3^
		5–6 days	Decrease	Decrease	Increase	
>P90 (adult)	WT	7 days	Decrease	n.s.	Increase	
	TNFα^−/−^	7 days	Decrease	n.s.	Increase	
	CaMKIIT286A	7 days	n.s.	n.s.	n.s.	
P28	PV-CRE:iA9	1 day occluding device	-	firing rates increase in ENs and decrease in INs		Kuhlman et al., 2013^4,5^
	WT	3days occluding device	Decrease	-	-	
	WT, Diazepam^*e*^		n.s.	-	-	
P35	PV-DREADD^*f*^ saline^*g*^		-	n.s.	n.s.	
	PV-DREADD^*f*^ CNO^*g*^		-	Decrease	n.s.	
P30	WT with SC	4 days	Decrease	Decrease	n.s.	Greifzu et al., 2014^3^
P25–35	WT with SC, PT lesion		Decrease	Decrease	n.s.	
P90	WT with SC	7 days	Decrease	n.s.	Increase	
P90	WT with EE, PT lesion	4 days	Decrease	Decrease	n.s.	
P130, 235	WT with SC	7 days	n.s.	n.s.	n.s.	
P130	WT with EE		Decrease	Decrease	n.s.	
P220	WT with EE		Decrease	Decrease	n.s.	
P250	WT with EE since P110		Decrease	n.s.	n.s.	
> P130	WT with SC, with RW since birth or 7 days during MD	7days	Decrease	Decrease	n.s.	Kalogeraki et al., 2014^3^
~P50	WT with SC, with 14 day DE, before MD	4 days	Decrease	n.s.	Increase	Stodieck et al., 2014^3^
~P110	WT with SC, with 14 day DE, before MD	7 days	Decrease	n.s.	Increase	
~P130	WT with SC, with 14 day DE, before MD, sham or PT lesion		Decrease	-	-	
~P136	WT with SC, with 14 day DE, before MD, Diazepam injection during MD		n.s.	n.s.	n.s.	
>P500	WT with SC, with 14 day DE before MD		Decrease	n.s.	n.s.	
>P60	Lynx1 KO	4 days	Decrease	Decrease	n.s.	Morishita et al., 2010; Bukhari et al., 2015^ 1^
	Lynx1 + tPA DKO		n.s.	-	-	
<P110	WT	7days	Decrease	n.s.	Increase	Huang X. et al., 2015^3^
>P110			n.s.	n.s.	n.s.	
<P110, >P110	PSD-95 KO	4, 7 days	Decrease	Decrease	n.s.	
P80–106	WT with Diazepam	7 days	n.s.	n.s.	n.s.	
P80–106	PSD-95 KO	7 days	Decrease	Decrease	n.s.	
>P80	PSD-95 KD at P0 or P40	4 days	Decrease	Decrease	n.s.	
P93–149	PSD-95 KO with PT lesion	7 days	Decrease	Decrease	n.s.	Greifzu et al., 2016^3^
P24–35	WT with EE	2 days	Decrease	Decrease	n.s.	Kalogeraki et al., 2017^3^
		4 days	Decrease	Decrease	n.s.	
		7 days	Decrease	Decrease	n.s.	
P90–104		4 days	Decrease	n.s.	Increase	
		7 days	Decrease	Decrease	n.s.	
P117–283		4 days	Decrease	n.s.	Increase	
		7days	Decrease	Decrease	n.s.	
>P200	WT with EE, transferred to SC, with regular water or fluoxetine	7days	n.s.	n.s.	n.s.	
>P200	WT with EE, transferred to SC, with RW	7days	Decrease	Decrease	n.s.	
P119–143	WT with long-term visual water maze training	39-44 days	Decrease	Decrease	n.s.	Hosang et al., 2018^3^
	WT with SC	47–50 days	Decrease	n.s.	n.s.	
P24–27, P28-35	WT	4 days	Decrease	Decrease	n.s.	Favaro et al., 2018^3^, also see Iwai et al., 2003^ 1^ dark reared WT animals express ODP beyond P40
P24–27	PSD-93 KO		Decrease	Decrease	n.s.	
P28–35	PSD-93 KO, NR or DR		n.s.	n.s.	n.s.	
	PSD-93 KD in V1 P0-1		n.s.	n.s.	n.s.	
>P40–50	WT		n.s.	n.s.	n.s.	
	WT, DR		Decrease	Decrease	n.s.	
P26	L4 GluN1 KO	3d, 7–8 days	n.s.	n.s.	n.s.	Fong et al., 2020^2^
P73		7–8 days	Decrease	n.s.	Increase	

*^#^Age of starting of MD; ^∗^lid suture, unless otherwise noted; ^a^contralateral bias index (CBI); ^b^ocular dominance index (ODI), ratio of responses of spared eye to non-spared eye; ^C^Diazepam infusion 1 day before and throughout 4 days of MD; ^d^daily Diazepam infusion postnatal days 23–30; ^e^Diazepam infusion immediately after MD for 2 days; ^f^PV-CRE with virally delivered DIO-hM4D DREADD and GCAMP6s; ^g^injection right after MD, 12 h interval for 24 h; n.s., not significant; –, not available; WT, wild type; MD, monocular deprivation; KO, knock out; CP, critical period; EN, excitatory neuron; IN, inhibitory neuron; EE, enriched environment; SC, standard cage; DE, dart exposure; NR, normal rearing; DR, dark rearing; KD, knock down; PT, photothrombotically induced; RW, running wheel; ^1^single unit recording; ^2^visually evoked potential; ^3^intrinsic signal optical imaging; ^4^two-photon calcium imaging; ^5^loose whole cell patch clamping in vivo.*

## Model of Critical Period Excitatory Neural Network Refinement

**FIGURE 1:**
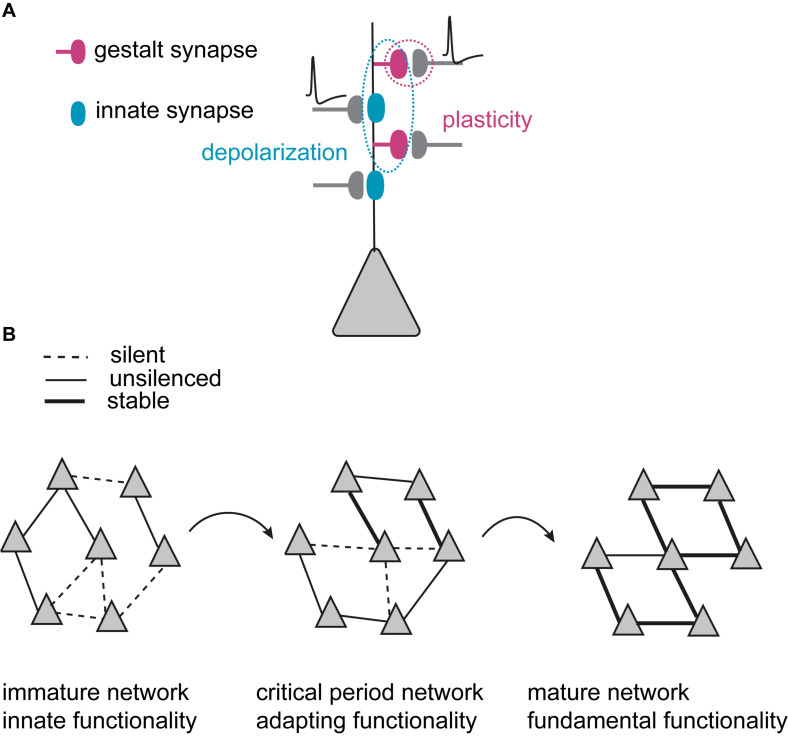
Model of critical period excitatory neural network refinement. **(A)** Two categories of synapses that are developmentally and functionally defined: ***Innate synapses*** (blue) that establish innate functionality of the network, and ***gestalt synapses*** (dark magenta) that primarily govern the plasticity and experience-dependent changes in network configurations during critical periods. Innate synapses provide depolarization (dotted blue circle), while plasticity happens at the gestalt synapses (dotted dark magenta circle). **(B)** Experience-dependent network maturation is mediated by synaptogenesis, gestalt synapse maturation and synapse pruning. Triangles illustrate principal neurons. During the critical period, the immature network starts with less specific connections. Through many iterations of synaptogenesis, maturation and pruning, the network becomes increasingly more organized with selectively strengthened, stable connections. The refined configuration (illustrated as symmetry) in the mature network builds the gained gestalt to represent the higher functionality acquired through experience. It is the gestalt that determines the network’s ability to present the sensory information as the percept.

We propose two categories of synapses that are developmentally and functionally defined: ***Innate synapses*** establish innate network functionality, and ***gestalt synapses*** primarily govern experience-dependent refinement of principal neuron connections during critical periods (Figure 1A and Table 2). The word “gestalt” (\gə-’stält\), is derived from German with the meaning: “An organized whole that is perceived as more than the sum of its parts” (Oxford University Press). It describes the functional role of these synapses after experience-dependent network maturation, namely providing a configuration of connections, a “gestalt,” that stores higher-order instructive information for future reference and modification, particularly the percept of the world created as a result of the individual’s early life experience.

**TABLE 2:** Terminology in critical period plasticity.

Term	Definition
Innate synapse	The synapse that is established prior to the critical period, which does not require PSD-95 for stable AMPAR incorporation. It might establish the initial rudimentary excitatory network, thus ensuring that neurons get depolarized.
Gestalt synapse	The synapse that governs the plasticity and changes in network configurations, which goes through several states categorized below, and requires PSD-95 for stable AMPAR incorporation.
Silent state	The nascent glutamatergic synapse that lacks transmitting AMPARs, and is occupied by NMDARs.
Unsilenced state	The synapse that is newly unsilenced through LTP during the critical period. AMPARs are newly incorporated into the synapse, and additional mechanisms are required for synapse stabilization. The synapse is prone to pruning by LTD.
Stable state	The synapse with stable AMPAR incorporation. Plasticity thresholds are increased and the synapse is unlikely to be pruned.
Excitatory synapse maturation	The process of experience-dependent strengthening and stabilization of excitatory synaptic connections during the critical period. It involves the unsilencing of silent synapses and stabilization of newly unsilenced synapses. The functional readout is the increase of synaptic potency, which is mediated by an increase in synaptic AMPARs.
Excitatory network maturation	The process of experience-dependent modification of the excitatory network through excitatory synapse maturation and experience-dependent synapse pruning. The functional architecture is modified to stably encode experience in the network. Both synapse maturation and synapse pruning decrease silent synapses as the substrate for network modifications. This maturation process terminates when the silent synapses diminishes.

By containing α-amino-3-hydroxy-5-methyl-4-isoxazole propionic acid-type glutamate receptors (AMPARs), innate synapses depolarize the postsynaptic membrane upon glutamate binding and mediate fast excitatory synaptic transmission in neuronal networks. During early development, innate synapses thus provide basic functionality by maintaining rudimentary excitatory network activity. The nascent gestalt synapses have an AMPAR-silent state (commonly referred to as silent synapses) (Isaac et al., 1995; Liao et al., 1995) and thus constitute morphological synaptic connections that transmit little or no information between the connected neurons. Nascent gestalt synapses are constantly formed throughout the critical period, creating random synaptic opportunities between principal neurons. The activation of innate synapses depolarizes neurons to enable associative plasticity at silent gestalt synapses that need coincidence of depolarization and presynaptic release at the silent gestalt synapses (Figure 1A).

Nascent (silent) gestalt synapses can, therefore, undergo experience-dependent ***excitatory synapse maturation*** during critical periods: repeated correlated neuronal activity of the pre- and postsynaptic neurons induces long-term synaptic potentiation (LTP), and drives AMPARs into silent gestalt synapses, transforming them into ***unsilenced*** gestalt synapses which now also contain responsive AMPARs (AMPAR^+^). These unsilenced gestalt synapses then get either stabilized to become ***stable*** gestalt synapses, consolidating a favorable synaptic connection, or get pruned if not timely and sufficiently activated. Thus, at the beginning of the critical period, the excitatory network consists of both rudimentary functional connections with innate synapses and gestalt synapses as potential substrates for experience-dependent network refinement. The coordinated action of unsilencing and stabilization of silent gestalt synapses, and pruning of gestalt synapses in the silent as well as the unsilenced state (but not stable gestalt synapses), underlies ***excitatory network maturation***. This coordinated process sets up the fundamental cortical network architecture (Figure 1B). We thus postulate that the network gains the gestalt that mediates its higher functionality via experience-dependent refinement for visual perception in adults. It is the gestalt that determines the network’s ability to present the sensory information as a percept.

## Silent Synapses as Substrates for Critical Period Plasticity

**FIGURE 2:**
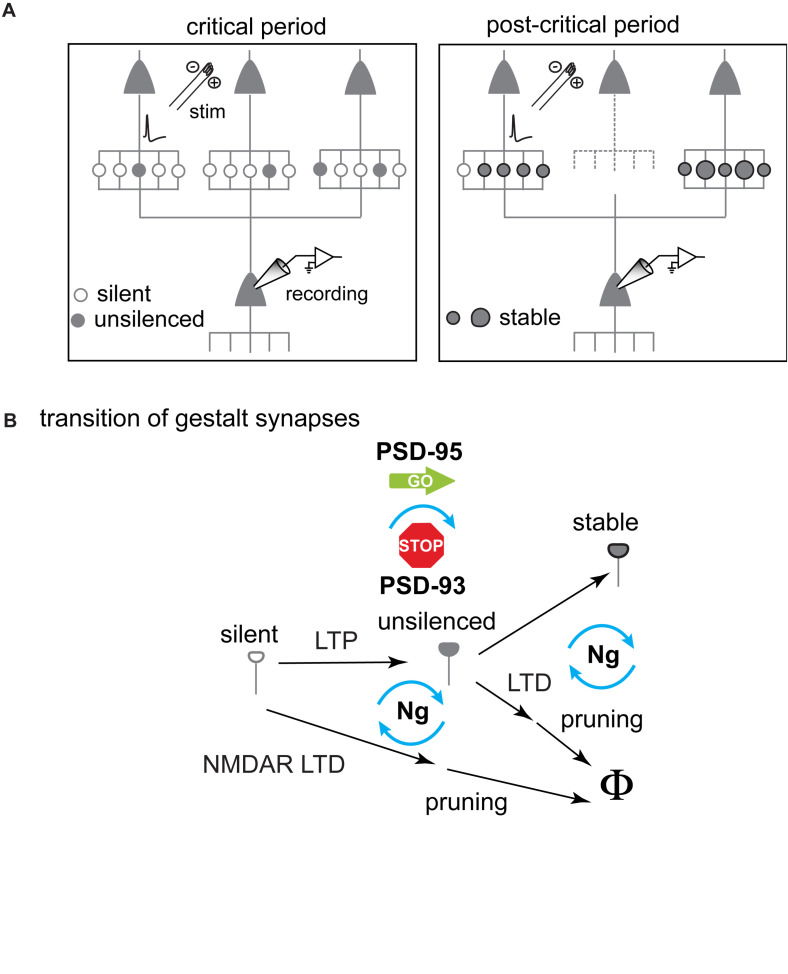
Schematic diagram illustrating the hypothesized changes of gestalt synapses underlying experience-dependent network maturation in the primary visual cortex during the critical period for ODP. **(A)** Increase of synaptic potency as the result of gestalt synapse maturation. Specifically, the number of stabilized synapses increases between certain neuron pairs. Gestalt synapses that are not stabilized are pruned away (dotted line). **(B)** The transition of different states of gestalt synapses. LTP drives AMPARs into silent gestalt synapses transforming them to ***unsilenced*** gestalt synapses now containing AMPARs (AMPAR^+^). These unsilenced gestalt synapses then get either stabilized to become ***stable*** gestalt synapses, consolidating a favorable synaptic connection, or get pruned if not timely and sufficiently activated. If not unsilenced, silent gestalt synapses are pruned away after NMDAR-LTD. Molecularly, PSD-95 drives the maturation of silent synapses, whereas PSD-93 opposes this function. Both maturation and pruning are likely controlled through post-synaptic calcium signaling, and neurogranin (Ng) is indicated as a key switch for governing the fate of gestalt synapses.

Silent synapses have so far been considered to represent a nascent state of functionally indistinguishable synapses. Here, we refer to silent synapses as the silent state of gestalt synapses. Postsynaptically silent synapses are nascent synapses that express *N*-methyl-D-aspartate-type glutamate receptors (NMDARs), but lack AMPARs (Isaac et al., 1995; Liao et al., 1995). At the resting potential, NMDARs are largely blocked by Mg^2+^. Hence at silent synapses, presynaptically released glutamate alone does not evoke an excitatory postsynaptic current (EPSC) in the recipient neurons when the recipient neurons sit at the resting potential. During LTP induction, NMDARs at silent synapses are opened with simultaneous postsynaptic depolarization and presynaptic glutamate release. Ca^2+^ influx through NMDARs drives Ca^2+^-dependent signaling cascades that lead to AMPAR incorporation into silent synapses, establishing AMPAR-mediated synaptic transmission (AMPAR positive; AMPAR^+^) that subsequently responds to presynaptic glutamate release at resting potential (Isaac et al., 1995; Liao et al., 1995). During the critical period, silent synapses mature, by recruiting and stabilizing AMPARs into the postsynaptic membrane, so that the proportion of silent synapses decreases with age and experience (Isaac et al., 1997; Bottjer, 2005; Funahashi et al., 2013; Huang X. et al., 2015; Han et al., 2017; Favaro et al., 2018). The functional consequence of this process is the increase of synaptic potency at unitary neural connections (Figure 2A; Isaac et al., 1997; Favaro et al., 2018). In the mature cortex, one excitatory axon forms on average five synapses with a target pyramidal neuron (Feldmeyer et al., 2002). The maturation of the gestalt synapses from an initially silent to an unsilenced and finally stabilized state, therefore, strengthens the connectivity of functional ensembles, representing a specific functional output of neural networks (Hebb, 1949). Thus, silent synapses constitute synapse opportunities, providing morphological (anatomical) connections between two neurons, and act as substrates to establish instructive connections when unsilenced and stabilized.

While functional ensembles are strengthened, the total number of excitatory synapses decreases during the critical period, and AMPAR-mediated synaptic transmission remains at equilibrium. The maturation of silent synapses is counter-balanced, at least in part, by synapse pruning (Han et al., 2017). This pruning is likely mediated by a form of long-term synaptic depression (LTD) (Nägerl et al., 2004; Zhou et al., 2004). Thus, maturation of silent synapses may consolidate a nascent synaptic connection, while its pruning eliminates the connection, resulting in a change of the connection pattern between excitatory neurons (Figure 2B). Consequently, silent synapse-based excitatory network maturation contains the essential ingredients for experience-dependent neural network refinement (Changeux and Danchin, 1976; Katz and Crowley, 2002). This excitatory network maturation process is reminiscent of the earlier work about the development of long-range, tangential connections in cat V1. At the beginning of the critical period, the V1 horizontal fiber network is rather homogeneously connected. Connections that participated in synchronized activities are stabilized (i.e., *neurons wire together if they fire together*) (Löwel and Singer, 1992) and network refinement includes pruning as well as a further elaboration of distinct connections (Callaway and Katz, 1990).

A critical role of silent synapses in juvenile ODP was revealed by the analysis of knock-out (KO) mice of postsynaptic density protein-93 (PSD-93) and PSD-95 (Huang X. et al., 2015; Favaro et al., 2018). PSD-93 and PSD-95 belong to the protein family of signaling scaffolds of the postsynaptic density that mediate signaling specificity in synaptic plasticity (Xu et al., 2008; Liu et al., 2017). PSD-95 is required for the experience-dependent maturation of silent synapses. A loss-of-function results in the persistent presence of a high fraction of silent synapses (∼50%) from eye-opening into adulthood in V1, as well as lifelong juvenile ODP (Huang X. et al., 2015). Additionally, when silent synapses are reinstated after the closure of the critical period by knocking down PSD-95 in the adult visual cortex, both the juvenile fraction of silent synapses and juvenile-like ODP are restored, indicating that PSD-95 is necessary for both the initial maturation of silent synapses and the maintenance of the matured synaptic state (Huang X. et al., 2015). Conversely, PSD-93 inhibits experience-dependent maturation of silent synapses (Favaro et al., 2018). In mice with a knock-out or visual cortex specific knock-down of PSD-93, silent synapses mature precociously, and consequently, the critical period for ODP closes earlier than that of wild-type mice. Notably, silent synapse maturation is delayed by dark rearing in wild-type mice and the duration of the critical period is consequently extended (Gianfranceschi et al., 2003; Funahashi et al., 2013; Favaro et al., 2018). However, in PSD-93 KO mice, dark rearing did not prevent the accelerated silent synapse maturation, nor the precocious termination of the critical period for ODP (Favaro et al., 2018).

The developmental increase in the protein expression of PSD-93 in V1 leads that of PSD-95 after eye-opening, and peaks before the critical period, whereas the expression of PSD-95 peaks at the end of the critical period, a result consistent with the opposing yet cooperative interaction of these paralogs in regulating silent synapse maturation (Huang X. et al., 2015; Favaro et al., 2018). The strict correlation between the time course of PSD-93 and PSD-95 expression, silent synapse maturation, and the duration of the critical period for ODP strongly imply that silent synapses serve as instructive substrates for network refinement during critical periods. While PSD-95 drives the maturation of silent synapses, PSD-93 opposes this function (Figure 2B; Favaro et al., 2018). The decrease of silent synapses may thus mark the end of the critical period, linking excitatory network maturation with the availability of silent synapses. Consistent with this notion, the accelerated loss of silent synapses also leads to early termination of the critical period in the primary auditory cortex (Sun et al., 2018). The opposing function of PSD-95 and PSD-93 in silent synapse maturation appears universal in gestalt synapses of principal neurons, as any of those synapses so far investigated, exhibit the same phenotype as described for layer 2/3 pyramidal neurons in V1 with ∼50% of synapses being regulated by these paralogs (Béïque et al., 2006; Huang X. et al., 2015; Shukla et al., 2017; Favaro et al., 2018).

Thus, under physiological conditions during the critical period, silent gestalt synapse-based refinement alters principal neuron wiring and their progressive maturation ends critical periods.

## Two Functionally Distinct Categories of Excitatory Synapses With Different Requirements on PSD-95 Function

The two different types of AMPAR^+^ synapses were indicated by the analysis of the role of PSD-95 in critical period plasticity. PSD-95 is not required for all stable AMPAR^+^ synapses. First, the excitatory drive onto PV^+^ interneurons is unaltered in PSD-95 KO mice (Huang X. et al., 2015), although PSD-95 is also expressed in PV^+^ interneurons and thus absent in the excitatory synapses of the mutant mice (Akgul and Wollmuth, 2010) indicating that the basal excitatory postsynaptic function in PV^+^ interneurons does not require PSD-95. Secondly, the maturational defect by loss of PSD-95 is limited to a subset of synapses in principal neurons. At eye-opening, in both WT and PSD-95 KO mice, ∼50% synapses are AMPAR^+^. Notably, at this developmental point, there is hardly any PSD-95 expressed in V1 (Huang X. et al., 2015). While in WT mice, PSD-95 protein levels increase and the fraction of silent synapses decreases after eye-opening, in PSD-95 KO mice, the fraction of silent synapses remains at ∼50%. Third, when knocking-down PSD-95 after silent synapses have matured, the fraction of silent synapses is restored to the level at eye-opening (∼50% synapses). The remaining ∼50% synapses are AMPAR^+^ synapses persisting in the absence of PSD-95, as we and others had observed in PSD-95 KO mice (Béïque et al., 2006; Huang X. et al., 2015). These results indicate that about half of the synapses onto principal neurons and all excitatory synapses onto PV^+^ interneurons do *not* require PSD-95 for synaptic AMPAR stabilization. We operationally define these PSD-95 *in*dependent synapses as *innate synapses*, which potentially provide constitutive excitatory drive onto neurons and build the rudimentary backbone network with innate function. In contrast, the PSD-95 dependent synapses are *gestalt synapses*.

The morphological identity of innate and gestalt synapses remains speculative. Dendritic spines are the typical morphological structures of glutamatergic synapses in the mature brain, categorized into thin, mushroom, and stubby spines (Juraska and Fifkova, 1979; Miller and Peters, 1981; Harris et al., 1992). Each spine generally contains one excitatory synapse, and hence the spine number has been widely used as a proxy for the number of glutamatergic synapses (LeVay, 1973; Harris et al., 1992). However, during early development, initial excitatory synapses are also formed onto the dendritic shaft of principal neurons of visual cortex, and other cortical and subcortical areas (Juraska and Fifkova, 1979; Miller and Peters, 1981; Boyer et al., 1998; Jang et al., 2015). Thus, shaft excitatory synapses might constitute part of the innate synapses. In layers 2/3 of the visual cortex of adult mice or rats, excitatory shaft synapses are rare, whereas stubby spines account for 20–30% of all spines (Miller and Peters, 1981; Majewska and Sur, 2003; Bukhari et al., 2015; Hodges et al., 2017). As the knock-down of PSD-95 revealed the presence of innate synapses in adult mice (Huang X. et al., 2015), we postulate that both shaft and stubby spines belong to the category of innate synapses. Based on dendritic compartmentalization theory, stubby synapses functionally resemble shaft synapses, as they are not electrically and morphologically separated from the dendritic shaft as the other types of spine synapses (Yuste, 2013).

Spine synapses start to form in the visual cortex after P7 in rats (Juraska and Fifkova, 1979; Miller and Peters, 1981) and the relative fraction of spine synapses increases during development (Harris et al., 1992). We postulate that synapses on thin and mushroom spines constitute the gestalt synapses that primarily govern the plasticity and changes in network configurations during critical periods. The observation that in some neurons, both shaft and spine synapses coexist throughout life, and that they do not convert into each other, further supports our two category concept with innate as shaft and gestalt as spine synapses, respectively (Reilly et al., 2011; Jang et al., 2015). This morphological differentiation might not be absolute and the main discriminator of the two synapse categories is the dependence of stable AMPAR incorporation by PSD-95.

PSD-95 mediates the maturation of silent gestalt synapses (Huang X. et al., 2015; Favaro et al., 2018) and thus the stable incorporation of AMPARs into gestalt synapses. This notion is further substantiated by the observations that first, loss of PSD-95 leads to ∼50% reduction in AMPAR EPSCs, corresponding to a reinstatement of ∼50% silent synapses (Nakagawa et al., 2004; Béïque et al., 2006; Elias et al., 2006; Schlüter et al., 2006; Ehrlich et al., 2007; Carlisle et al., 2008; Xu et al., 2008; Krüger et al., 2013) second, the increase in PSD-95 protein levels during the critical period; and third, gain of PSD-95 function increases AMPAR EPSCs by accelerated silent synapse maturation in developmentally young but not adult brains (Stein et al., 2003; Levy and Nicoll, 2017; Favaro et al., 2018).

The hypothesized two functional categories of synapses become apparent also in other KO mice. Loss of cadherin, EGF LAG seven-pass G-type receptor 3 (Celsr3) leads to a loss of ∼50% glutamatergic synapses (Thakar et al., 2017) a result consistent with our two synapse categories concept, if one of them strictly requires Celsr3. Furthermore, loss- and gain-of-function of the signaling scaffold membrane-associated guanylate kinase inverted-2 and atrophin interacting protein-1 (MAGI-2) leads to a shift in the fraction of stubby spines and mushroom spines (Danielson et al., 2012). Further analyses of the functional and molecular interactions between PSD-95 and Celsr3 or MAGI-2, respectively, might reveal more insights about the identity and function of these synaptic categories.

Thus, both morphological as well as functional characterizations, support at least two synaptic categories. Given that PSD-95 is expressed in shaft synapses of PV^+^ interneurons, the differentiation is not possible by the sole presence or absence of PSD-95, but might be rather determined by one of the PSD-95 interaction proteins, such as Celsr3 (Thakar et al., 2017).

## Interaction of Synaptogenesis, Synapse Maturation, and Synapse Pruning in Critical Period Plasticity

Based upon studies investigating dendritic spines, the morphological proxy of excitatory synapses, we postulate that nascent gestalt synapses are constantly generated during the critical period and serve as synaptic substrates for experience-dependent refinement by being unsilenced and stabilized, or pruned. Consistent with our model, the density of dendritic spines in the cerebral cortex peaks during early childhood, and then slowly decreases toward adulthood in humans, non-human primates, and other mammalian brains (Huttenlocher, 1979; Juraska and Fifkova, 1979; Rakic et al., 1986; Munoz-Cueto et al., 1991; Bourgeois and Rakic, 1993; Huttenlocher and Dabholkar, 1997).

Previous models have focused on the motility of filopodia and spines in mediating structural plasticity during critical period network refinement (Ziv and Smith, 1996; Dunaevsky et al., 1999; Lendvai et al., 2000). Filopodia are thin, long protrusions of the dendritic shaft that form and disappear in minutes both in cultured neurons and *in vivo* (Dailey and Smith, 1996; Ziv and Smith, 1996; Lendvai et al., 2000). They lack postsynaptic specializations and are postulated to sample the surrounding for new synaptic connections (Ziv and Smith, 1996). Filopodia are particularly abundant during early development; in layer 2/3 of the visual cortex, they are evident between P3 and P12 (Miller and Peters, 1981). The number of filopodia decreases to a low level at the beginning of the critical period in visual cortex (Majewska and Sur, 2003; Konur and Yuste, 2004; Cruz-Martin et al., 2010). Sensory experience drives the motility of dendritic protrusions in dendrites of layer 2/3 pyramidal neurons in the somatosensory cortex during critical periods (Lendvai et al., 2000). However, dark rearing which prolongs the critical period for ODP does not prolong the time window of highly motile spines/filopodia in the visual cortex (Konur and Yuste, 2004). Hence, interrogations that extend critical periods and the motility of spines/filopodia appear disconnected.

Furthermore, the high numbers of filopodia in early postnatal (P3–P12) cortex is overlapping with the critical period of the somatosensory cortex (P11–13), but not with the later critical period in the visual cortex (P21–35) when filopodia numbers are low (Miller and Peters, 1981; Dunaevsky et al., 1999; Lendvai et al., 2000). While it is conceivable that filopodia are responsible for synapse increase before the critical period (Juraska and Fifkova, 1979; Munoz-Cueto et al., 1991), they are unlikely to play a major role for synaptogenesis during the critical period, at least in the visual cortex (Majewska and Sur, 2003; Konur and Yuste, 2004; Cruz-Martin et al., 2010). Nascent gestalt synapses in the visual cortex might thus rather be formed as thin spines that are unstable when lacking PSD-95, and that mature with PSD-95 incorporation (Cane et al., 2014).

While synaptogenesis already starts prenatally, the increase of synapse numbers continues postnatally, providing evidence for postnatal synaptogenesis (Huttenlocher, 1979; Juraska and Fifkova, 1979; Rakic et al., 1986). Postnatal synaptogenesis is also supported by more recent spine imaging studies using *in vivo* two-photon microscopy (Grutzendler et al., 2002; Trachtenberg et al., 2002). Spine formation is prominent in juvenile rodents during the critical period for ODP in V1 (Grutzendler et al., 2002; Holtmaat et al., 2005; Zhou et al., 2017) and also in somatosensory, motor and frontal cortices (Lendvai et al., 2000; Holtmaat et al., 2005; Zuo et al., 2005) likely replenishing the nascent gestalt synapse pool for further network refinement. However, little is known whether synaptogenesis or its kinetics are regulated by experience during the critical period.

Synapse elimination has been widely reported in the neuromuscular junction, the ganglia of the peripheral nervous system, cerebellum, and spinal motor neurons (Purves and Lichtman, 1980; Colman et al., 1997). Eliminating underutilized connections to refine the network structure has long been hypothesized to be the leading mechanism in the development of both the peripheral and central nervous systems (Shatz and Stryker, 1988; Simon et al., 1992; Walsh and Lichtman, 2003). At both the neuromuscular junction and the retino-thalamic connections, in a “*winner takes it all*” process, one neuron typically wins the competition among a group of neurons to connect to the target neuron/muscle, while the other connections are pruned (Kasthuri and Lichtman, 2003; Hooks and Chen, 2007).

The synaptic reorganization in the cortex appears to be different, not the least because neurons are innervated by thousands of afferents in complex dendritic trees. MD increases the structural dynamics of spines (Tropea et al., 2010; Yu et al., 2011) and accelerates spine elimination during the critical period in V1 (Yu et al., 2011; Zhou et al., 2017; Sun et al., 2019), indicating that pruning also contributes to experience-dependent structural plasticity in the cortex. However, in the cerebral cortex, the wiring configuration among principal neurons is more complex and silent gestalt synapses might rather operate under a process, subsumed as “*use it or lose it*,” a concept, theorized for bidirectional plasticity such as LTP and LTD (Hebb, 1949; Miller et al., 1989; Balice-Gordon and Lichtman, 1994; Katz and Shatz, 1996). In contrast to LTP and LTD, not only synaptic weights are changed during experience-dependent plasticity, but synaptic connections between principal neurons are gained with synaptogenesis and silent synapse maturation, or lost with synapse pruning.

While little is known about the pruning of (silent) gestalt synapses, their partly transient nature is indicated by the observation that the rate of dendritic spine turn-over is high during early cortical development and in PSD-95 deficient neurons, situations when silent synapse numbers are high (Zuo et al., 2005; Ehrlich et al., 2007). Given the equilibrium of AMPAR-mediated synaptic transmission during critical periods (Han et al., 2017), the model proposes that some AMPAR^+^ connections are weakened by uncorrelated neuronal activities, resulting in synaptic depression and ultimately spine pruning. In contrast, if repeatedly and successfully activated by experience, silent synapses may be unsilenced and consolidated into mature synapses between principal neurons to improve network function.

Recent findings provided new insights on mechanisms coordinating synapse maturation and synapse pruning in neurons. Neurogranin is a Ca^2+^/calmodulin (CaM) binding protein that regulates the availability of the Ca^2+^ signal mediator CaM and consequently regulates the activation of the downstream signaling proteins including CaM-dependent phosphatase and CaM-dependent kinase. Tipping the Ca-dependent signaling toward CaM-dependent phosphatase activation by genetically reducing or abolishing the function of neurogranin (Hwang et al., 2018), hinders the maturation of silent gestalt synapses and exacerbates synapse pruning (Han et al., 2017). Thus, both maturation *and* pruning are likely controlled through post-synaptic calcium signaling, and neurogranin is indicated as a key switch for governing the fate of gestalt synapses (Figure 2B).

Notably, both synaptogenesis and synapse pruning are also strongly influenced by astrocytes and microglia, reviewed by Clarke and Barres (2013); Chung et al. (2015), and Neniskyte and Gross (2017). While many astrocyte-secreted factors have been reported to drive synaptogenesis *in vitro* in the peripheral or central nervous systems (reviewed by Clarke and Barres, 2013), existing data support the involvement of thrombospondins in driving excitatory synaptogenesis in the cortex (Christopherson et al., 2005; Eroglu et al., 2009), and silent synapse generation after cocaine in the nucleus accumbens (Wang et al., 2020). Studies primarily from the retinogeniculate system and hippocampus implicate that glial cells are involved in synapse elimination (Paolicelli et al., 2011; Schafer et al., 2012), mediated via the complement pathway of microglia (Stevens et al., 2007; Schafer et al., 2012), and via Multiple Epidermal Growth Factor-like Domains 10 (MEGF10) and MER receptor tyrosine kinase (MERTK) phagocytic pathways of astrocytes (Chung et al., 2013). It is not known whether these pathways also contribute to the experience-dependent maturation of gestalt synapses and if so whether there exists any synapse subtype specificity, concerning the neuro-immune interaction and communication to control the highly specific network refinement. A major outstanding challenge, therefore, remains to identify the subtypes of synapses by e.g., identifying molecular markers for categorizing synapses, notwithstanding their functional categorization in governing refinement processes of cortical networks.

According to our model, gestalt synapses are the primary source for experience-dependent plasticity during critical periods, especially for the experience-dependent strengthening of functional ensembles. Nevertheless, innate synapses also appear to be targets of regulation. This prediction is based on two results: First, the same fraction of AMPAR^+^ and silent synapses was found after later loss of PSD-95 and at eye-opening (Huang X. et al., 2015), indicating that the fraction of innate synapses remains constant with age. Second, the spine density decreases toward the end of the critical period, indicating a decrease in total numbers of synapses (Han et al., 2017). To keep the same share of innate synapses, both innate synapses and gestalt synapses must be pruned during development at a similar rate. These observations also predict that both categories of synapses might be homeostatically linked despite their different requirements on PSD-95, so that changes in the number of gestalt synapses influence the number of innate synapses.

Thus, at the beginning of the critical period, the excitatory network starts with both innate synapses and silent gestalt synapses. Innate synapses provide the activity to maintain a backbone of rudimentary excitatory neural network activity, and silent gestalt synapses provide potential substrates for experience-dependent network refinement. Additional nascent gestalt synapses are continuously formed during the critical period, setting up repeating iterative cycles for changing and optimizing neural network configurations. The excitatory network matures based upon the coordinated action of unsilencing and stabilization of silent gestalt synapses, and pruning of gestalt synapses in the silent as well as the unsilenced state, but not stable gestalt synapses. This coordinated process sets up the fundamental functional cortical network architecture (the gestalt) during the critical period (Figure 1). We hypothesize that this silent synapse-based network maturation during critical periods is universal for the cerebral cortex. In addition to the visual cortex, it also takes place in the auditory cortex (Sun et al., 2018), the somatosensory cortex (Isaac et al., 1997), the medial prefrontal cortex, and the hippocampus of rodents (Favaro et al., 2018). Silent synapses have also been shown to be necessary for experience-dependent network development/maturation beyond the mammalian brain, namely for song learning in zebra finches during the sensitive period (Bottjer, 2005).

## Silent Synapse-Based Excitatory Network Maturation Versus Synaptic Plasticity

This silent synapse-based mechanism of critical period plasticity is consistent with the heightened excitatory synaptic plasticity during this time window. The high susceptibility of the visual cortex for LTP coincides with the critical period and can be prolonged by dark rearing (Kirkwood et al., 1995), which prevents silent synapse maturation (Funahashi et al., 2013; Favaro et al., 2018). Unsilencing silent synapses is a mechanism for LTP, especially at a young age (Isaac et al., 1995), thereby strengthening and stabilizing functional connections by increasing the number of AMPAR^+^ synapses at these connections. Similar to the increase in potency by silent synapse maturation in V1, LTP increases the potency in minimal stimulation recordings, consistent with the hypothesis that LTP mechanisms mature silent synapses *in vivo* (Liao et al., 1995; Isaac et al., 1996, 1997; Favaro et al., 2018).

The progressive maturation of silent gestalt synapses predicts that the number of AMPAR^+^ synapses should increase during development. However, the frequency of miniature (m) EPSCs remains unchanged throughout the critical period until adulthood (Corlew et al., 2007; Han et al., 2017). This equilibrium is at least partially explained by the observation that some existing AMPAR^+^ synapses go through experience-dependent elimination and thus counterbalance excitatory synapse maturation during the critical period, as spine density, the anatomical proxy of excitatory synapses also decreases during the critical period (Han et al., 2017). Mechanistically, this pruning is likely mediated by a form of LTD. In support of this notion, it was shown that synapse pruning results from a form of LTD (Nägerl et al., 2004; Zhou et al., 2004), and lowering the threshold for LTD also leads to a profound spine loss (Han et al., 2017).

While reducing neurogranin levels lowers the threshold for LTD (Han et al., 2017) and blocks LTP (Pak et al., 2000; Hwang et al., 2018), enhancing neurogranin levels lowers the threshold for LTP (Zhong and Gerges, 2012; Hwang et al., 2018). Thus, changes in neurogranin levels likely lead to a form of metaplasticity by sliding the plasticity threshold (Bienenstock et al., 1982; Abraham and Bear, 1996), which in turn will influence critical period ODP. According to both the metaplasticity theory (Abraham and Bear, 1996; Philpot et al., 2001) and the silent synapse mechanism, we predict that lowering neurogranin levels will extend the critical period for ODP while enhancing neurogranin levels will drive a faster maturation of the cortical circuit, and likely drive the adult form of plasticity earlier. Testing how changes in neurogranin levels affect ODP will likely provide more insight into this process.

It should be noted that both synapse stabilization and synapse pruning are cellular events lasting beyond the time scale of the initial NMDAR-dependent bi-directional plasticity, and thus likely depend on additional trophic factors which initiate inter-spine competition that will ultimately determine synapse fate (maturation or pruning) (Bian et al., 2015).

## The Expression of Critical Period Ocular Dominance Plasticity

Before discussing how the concept of gestalt synapses integrates into the expression mechanisms of critical period ocular dominance plasticity, we review the current state of the art. MD shifts the ocular dominance of visual responses in the binocular segment of V1 toward the non-deprived eye. During the critical period, this shift is initially mediated by a depression of deprived-eye responses in cortical layer 4 and layer 2/3, measured with visually evoked potentials (VEPs) or calcium imaging, respectively (Box 1) (Frenkel and Bear, 2004; Mrsic-Flogel et al., 2007; Gandhi et al., 2008) or in superficial layers with intrinsic signal optical imaging (Cang et al., 2005; Kaneko et al., 2008a, b). When analyzing synaptic strength directly with *in vivo* patch-clamp recordings of layer 4 spiny stellate neurons in the binocular region, excitatory synaptic inputs are primarily driven by visual stimulation of the contralateral eye in rodents, and the ocular dominance shift resembles that measured with VEPs (Ma et al., 2013). The rapid ocular dominance shift initiated by 3–4 days of MD in juvenile rodents is primarily expressed at the *thalamocortical* glutamatergic feedforward synaptic connection, as pharmacological removal of *intracortical* polysynaptic inhibitory and excitatory synapses did not influence the magnitude of the ocular dominance shift (Khibnik et al., 2010). Despite the proposed expression of ODP at the glutamatergic synapses, plasticity in inhibitory neurons is expressed in parallel. The strength of the feed-forward inhibitory drive of either pathway is equilibrated to the excitatory drive, resulting in a similar excitatory/inhibitory ratio driven by either eye, echoing equilibration in layer 2/3 pyramidal neurons (Xue et al., 2014). MD causes a depression of the excitatory drive selectively of the deprived eye pathway, while the feed-forward inhibition of both deprived and non-deprived eye pathways is reduced. The resulting shift in the response of the layer 4 spiny stellate neurons is thus mediated by both a shift in the excitatory drive toward the non-deprived eye and a relative decrease of inhibition of the non-deprived eye drive (Ma et al., 2013). This reduction of the feed-forward inhibition of both pathways is also seen in layer 2/3 (Kuhlman et al., 2013), enabling the expression of critical period ODP. The depression of the excitatory synaptic drive reaches a maximum after 3-d MD. In contrast, the depression of feed-forward inhibition progresses further and after 6-day MD, the excitatory/inhibitory ratio of the non-deprived eye inputs is higher than that of the deprived contralateral eye input (Ma et al., 2013).

Mechanistically, multiple lines of evidence point toward NMDAR-dependent LTD to cause deprived eye depression (Bear et al., 1990; Rittenhouse et al., 1999; Sawtell et al., 2003; Yoon et al., 2009). First, NMDAR-dependent LTD is prevalent during critical periods, and second, MD occludes subsequent LTD *ex vivo* in visual cortex slices (Crozier et al., 2007). Third, the susceptibility of some forms of LTD declines in an age-dependent manner (Dudek and Friedlander, 1996; Kirkwood et al., 1997; Jiang et al., 2007), consistent with the absence of deprived eye depression in adult ODP of rodents raised in standard cages. Fourth, layer 4 activity is only depressed if the deprived eye sends spontaneous activity. If this activity is blocked by the sodium channel blocker tetrodotoxin, only the increase of the non-deprived eye pathway after longer MD is expressed (Frenkel and Bear, 2004). This result is consistent with the dependence of LTD on unsynchronized neural activity, rather than lack of activity, between pre- and postsynaptic neurons to induce NMDAR activity. Fifth, MD induces a phosphorylation pattern of AMPARs that resembles the one after LTD induction (Heynen et al., 2003). Sixth, deprived eye depression only occurs in neurons receiving synaptic inputs from both eyes and requires the competing input of both eyes (Rittenhouse et al., 1999; Frenkel and Bear, 2004; Mrsic-Flogel et al., 2007), indicating that during MD, the spontaneous activity of the deprived eye is likely unsynchronized with the visually evoked non-deprived eye inputs so that depression is induced at the deprived eye inputs. Pruning of synapses that occurs in both layer 1 and layer 2/3 after short MD was observed in the binocular region of V1, indicating that LTD is a step preceding pruning or even part of it (Mataga et al., 2004; Yoon et al., 2009; Zhou et al., 2017; Fong et al., 2020).

Despite the canonical feedforward information flow of sensory stimuli from the thalamus via layer 4 spiny stellate neurons to layer 2/3 pyramidal neurons (Gilbert and Wiesel, 1979; Constantinople and Bruno, 2013), critical period ODP is expressed independently in each layer (Trachtenberg et al., 2000; Liu et al., 2008). Notably, depression in layer 2/3 occurs before depression in layer 4 (Trachtenberg et al., 2000). The mechanisms underlying LTD also differ in each layer. While low-frequency stimulation-induced LTD in both layer 4 and layer 2/3 depends on postsynaptic NMDARs, LTD in layer 4 is mediated by AMPAR endocytosis, whereas LTD in layer 2/3 is *in*dependent of AMPAR endocytosis but dependent on endocannabinoid receptor 1 (CB1R) activation (Rao and Daw, 2004; Crozier et al., 2007). When blocking LTD and ODP in layer 2/3 by CB1R blockers, ODP in layer 4 is still expressed (Liu et al., 2008) indicating that ODP in layer 2/3 is not a prerequisite for ODP in layer 4, even though layer 4 plasticity lags layer 2/3 plasticity. This independence might be partly caused by direct innervation of layer 2/3 from the thalamus, as demonstrated in other primary sensory cortices in mice (Petreanu et al., 2009).

A longer duration MD during the critical period (≥ 7 days) leads to a delayed increase of V1-responses to the non-deprived eye (Frenkel and Bear, 2004; Sato and Stryker, 2008) a phenomenon hypothesized to be due to homeostatic regulation after a prolonged decrease of deprived eye inputs (Kaneko et al., 2008b; Smith et al., 2009; Espinosa and Stryker, 2012; Ranson et al., 2012). Thus, depending on the duration of MD, interdependent mechanisms operate at different stages in the binocular region of the primary visual cortex to reorganize cortical neuron connectivity.

## The Apparent Paradox: Silent Synapses as Substrates for Critical Period Plasticity, and LTD as the Mechanism for Critical Period ODP

How do gestalt synapses integrate into the LTD-based expression mechanism of ODP? On the one hand, the presence of silent synapses defines the duration of the critical period for ODP (Huang X. et al., 2015; Favaro et al., 2018), and on the other hand, LTD is a mechanism of deprived eye depression in ODP, expressed by AMPAR endocytosis at least in layer 4 (Crozier et al., 2007; Yoon et al., 2009). These two concepts of mechanisms underlying critical period ODP appear paradoxical, as silent synapses lack AMPARs, which apparently conflicts with their direct involvement as substrates of LTD for critical period ODP. Additionally, with PSD-95 deficiency, silent synapses prevail and critical period ODP lasts lifelong, while LTD, as tested in the hippocampus, is impaired, a finding that also appears to be at odds with the role of LTD in critical period ODP (Carlisle et al., 2008; Xu et al., 2008; Huang X. et al., 2015). In fact, in PSD-95 KO mice, the threshold for LTP in the hippocampus is lower compared to wild-type mice (Migaud et al., 1998; Béïque et al., 2006; Carlisle et al., 2008). While this observation is consistent with the role of silent synapses as substrates for LTP (Isaac et al., 1995; Liao et al., 1995), it appears at odds with the prevailing high fraction of silent synapses in adult PSD-95 KO mice. If LTP is easier to achieve, why aren’t there more and stronger AMPAR^+^ synapses instead of silent synapses in adult PSD-95 KO mice? Conversely, in PSD-93 KO mice, silent synapses mature precociously and the critical period for ODP closes earlier, whereas the threshold for LTP in the hippocampus is reported to be elevated (Carlisle et al., 2008; Favaro et al., 2018). If LTP is harder to achieve, why aren’t there more silent synapses in adult PSD-93 KO mice? Thus, it appears that there is a paradox between the prevalence of silent synapses as substrates for ODP and LTD as the mechanism for ODP, as well as between the pace of maturation of silent synapses and the susceptibility of LTP in PSD-95 and PSD-93 KO mice.

At first sight, one might ask for additional experiments, as e.g., the mechanism of LTD underlying ODP in V1 layers 2/3 is distinct from NMDAR-dependent LTD in the hippocampus (Crozier et al., 2007; Xu et al., 2008). It is not known whether LTD in layer 2/3 is impaired in PSD-95 KO mice. While further studies are needed to fully resolve the paradox, some plausible predictions already unravel most of it. One key prediction drawn from the current literature that helps disentangle this apparent paradox is that the functional maturation of gestalt synapses processes through two steps, first unsilencing and then stabilization of synaptic AMPARs to consolidate a connection between two principal neurons, and the MD-induced LTD may happen at the unsilenced, but not stable gestalt synapses.

This two steps maturation process is implicated by studies of PSD-95 KO mice. In PSD-95 KO mice, the threshold for LTP in the hippocampus is low (Béïque et al., 2006; Carlisle et al., 2008). Consequently, memory acquisition in PSD-95 KO mice is normal or even better compared to WT mice (Fitzgerald et al., 2015; Shukla et al., 2017). However, memory retention is impaired. This disconnection can be explained as follows: NMDAR-dependent LTP drives the unsilencing of silent gestalt synapses in the hippocampus, and likely also in the visual cortex (Isaac et al., 1995; Kirkwood et al., 1995; Liao et al., 1995). The prevalence of silent gestalt synapses leads to more substrates for unsilencing, contributing to the lower threshold for LTP. However, the unsilenced gestalt synapses in PSD-95 KO mice are apparently not stable and AMPARs driven into them do not persist for longer time periods, as the fraction of silent synapses does not change throughout development. Furthermore, removing PSD-95 in adults will reinstate silent gestalt synapses (Huang X. et al., 2015). Thus, it is conceivable that silent gestalt synapses are initially converted into the unsilenced state, i.e., incorporate AMPARs into the postsynaptic membrane, but only transiently. The second step during gestalt synapse maturation, namely the stabilization of postsynaptic AMPARs, requires PSD-95.

**FIGURE 3:**
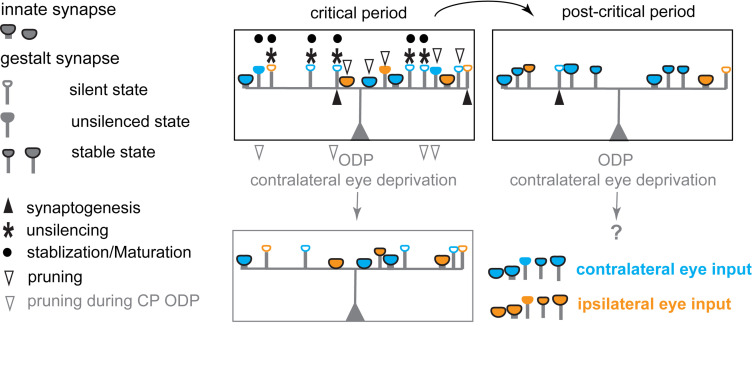
Schematic diagram illustrating the hypothesized changes in eye specific inputs underlying experience-dependent plasticity in the primary visual cortex during the critical period for ODP. During the critical period, postsynaptic neurons in binocular V1 receive inputs from both eyes with a strong overrepresentation of contralateral eye inputs, via both innate synapses and silent, and unsilenced gestalt synapses. Unsilencing of silent gestalt synapses, synaptogenesis, and pruning are ongoing: innate synapses, silent gestalt synapses, unsilenced gestalt synapses and newly generated silent gestalt synapses are present. After the critical period (post-critical period), some previously unsilenced gestalt synapses are consolidated, while others are pruned away, stabilizing the synapses continuously receiving correlated inputs after unsilencing. Contralateral eye deprivation drives depression and preferential pruning of deprived eye inputs, causing an OD-shift toward the open/non-deprived/experienced, ipsilateral eye.

During the critical period, some AMPAR^+^ synapses are thus unsilenced gestalt synapses, in an AMPAR^+^, but yet transient state. While both unsilencing and stabilization steps require correlated neural activity to drive LTP signaling pathways, at this stage, any uncorrelated neuronal activity can drive unsilenced gestalt synapses to depression, followed by pruning, so that the connection is lost during refinement (Figure 3). It is the very nature of the critical period that the network remains plastic until the most favorable configuration of the excitatory network is achieved. Thus, the prevalent unsilenced gestalt synapses that still need rounds of evaluation, and are vulnerable to depression, are the substrates for LTD. Depression is mediated by LTD or depotentiation, which are mechanistically similar, and will eventually lead to the pruning of some of the unsilenced AMPAR^+^ gestalt synapses. During MD, the lack of correlated activity in afferents of the deprived eye prevents stabilization of gestalt synapses, and thus favors the depression and pruning of unsilenced gestalt synapses, ultimately leading to the depression of the deprived eye inputs. This process is hypothesized to be unique to the critical period, most likely due to the prevalence of silent and unsilenced gestalt synapses that are not yet stabilized. Both processes, stabilization, and pruning of gestalt synapses, collectively lead to the decrease of silent synapses, and thus the decrease in the capability for plastic changes in mature, adult networks using this mechanism. A revealing test for this prediction will be the preservation of an LTD or depotentiation mechanism in the visual cortex of adult PSD-95 KO mice, presumably coupled with synapse pruning. Consistent with this prediction, the turnover of spines in the hippocampus of PSD-95-deficient neurons is elevated (Ehrlich et al., 2007).

Studies on the role of PSD-93 in plasticity were performed primarily in the hippocampus, and at adult ages (4–12 months) when developmental processes have ceased and plasticity follows different rules. In PSD-93 KO mice, LTP with a classical high-frequency train is similar to that in WT mice. Only with non-classical conditioning stimuli with 5 Hz, a reduction in potentiation in PSD-93 KO mice emerges (Carlisle et al., 2008). However, the mechanism of this form of potentiation was not further explored. Given the presence of inhibitory transmission, a distinction between disinhibition and potentiation cannot be made at present. A direct role of PSD-93 in developmental LTP for unsilencing silent synapses needs further research. Considering the precocious maturation of silent synapses in PSD-93 KO/KD mice (Favaro et al., 2018), PSD-93 more likely inhibits both the unsilencing of silent gestalt synapses by LTP and the stabilization of unsilenced gestalt synapses during early development.

Our survey of the apparent paradox about the mechanisms of critical period plasticity elucidates that synapses may go through stepwise transitions during the critical period, from silent gestalt synapses to unsilenced gestalt synapses that then either get stabilized or depressed and ultimately pruned if not further stabilized (Figure 2B). Which of the different possible scenarios happens depends on Hebbian plasticity, and the whole process goes beyond the time window of LTP and LTD (Figure 2B). During these stepwise transitions, discrete molecular events will take place, orchestrated via signaling scaffold proteins and calcium signaling. These molecular events include the insertion and stabilization of AMPARs, the exchange of NMDAR subtypes (Quinlan et al., 1999; Philpot et al., 2001; Bellone and Nicoll, 2007), and likely also the dynamic changes of synaptic scaffold composition that eventually determine the fate of a particular synapse. Although some key players that control the signaling scaffolds and the signaling cascade have already been identified, the overall molecular landscape regulating the process remains elusive. Further studies are required to identify the critical pathways and players, and to elucidate the exact sequence of molecular events leading to network maturation during critical periods.

## The Interplay of Local Inhibition and Silent Synapses During Critical Periods

While we propose that the expression of ODP is primarily mediated by excitatory gestalt synapses, the opening of the critical period is likely primarily mediated by the maturation of local inhibitory circuits in V1 (Hensch et al., 1998; Fagiolini and Hensch, 2000). The fraction of silent gestalt synapses among all synapses does not peak at the beginning of critical periods. In the rodent visual cortex, their fraction is highest at, or even before eye-opening (Rumpel et al., 2004; Funahashi et al., 2013; Favaro et al., 2018) while the critical period for ODP in V1 begins at P21 (Espinosa and Stryker, 2012). Hence, the sole appearance of silent gestalt synapses provides substrates for critical period plasticity but does not open critical periods. The opening of the critical period requires the maturation of the inhibitory circuit (Hensch et al., 1998; Fagiolini and Hensch, 2000), whereas the duration of the critical period is dictated by the availability of silent gestalt synapses (Huang X. et al., 2015; Favaro et al., 2018). With the beginning of the critical period in the visual cortex, the potency of minimally evoked synaptic responses increases, indicating excitatory synapse maturation by AMPAR incorporation (Favaro et al., 2018). However, the fraction of silent synapses decreases already before the onset of the critical period. Given that the mEPSC frequency increases steeply before the critical period while the synaptic potency doesn’t (Desai et al., 2002; Corlew et al., 2007; Han et al., 2017; Favaro et al., 2018), we postulate that unsilencing silent synapses before critical periods enhances the overall functional connectivity in the cortical network, whereas unsilencing silent synapses during the critical period increases the weight of connectivity.

The opening of the critical period for ODP in rodent V1 is marked by a steep increase in the expression of α1 type of GABA-A receptors at PV^+^ interneuron-innervated postsynaptic sites of pyramidal neurons (Klausberger et al., 2002; Fagiolini et al., 2004; Huang X. et al., 2015). Theoretic models imply that lateral inhibition is important for stimulus selectivity (Nass and Cooper, 1975), which is required for MD induced changes in cortical networks, as laid out in the BCM theory for cortical plasticity (Bienenstock et al., 1982). Consistent with this model, intracortical blockade of GABA transmission in the kitten striate cortex lowered orientation selectivity in neuronal responses, accompanied by abnormal receptive field properties and reduced ocular dominance shifts (Ramoa et al., 1988). Reduced GABAergic transmission as seen in the GAD65 knock-out mice correlates with the lack of the opening of the critical period for ODP (Hensch et al., 1998). The correlation between the maturation of the inhibitory system and the opening of the critical period for ODP is also seen in genetic models, targeting specific regulatory genes and pharmacological interrogations. Interventions that accelerate inhibitory system maturation often lead to a precocious opening of critical periods, whereas interrogations that delay inhibitory system maturation, delay the opening of critical periods, as has been reported for both the visual and auditory cortex (Hensch, 2005; Espinosa and Stryker, 2012; Levelt and Hübener, 2012; Takesian et al., 2018). The maturation of the inhibitory network is initiated by inhibitory neurons of the 5-HT_3A_R positive subgroup by Neuregulin/ErbB4 signaling (Sun et al., 2016; Takesian et al., 2018) while the maturation of the strength of α1-type GABA-A receptor-positive synapses, presumably from PV^+^ interneurons to pyramidal neurons, may initiate the onset of the critical period for ODP (Fagiolini et al., 2004). Diazepam, a positive allosteric modulator of GABA-A receptors, rescues the ODP deficit in GAD65 knock-out mice (Hensch et al., 1998) and is sufficient to initiate a premature opening of the critical period for ODP in WT mice (Fagiolini and Hensch, 2000; Chen et al., 2014). These results indicate that mechanisms for critical period cortical plasticity are already in place without proper inhibitory transmission and that the maturation of the inhibitory network likely opens the critical period for ODP. As a result, the maturation of the inhibitory system may allow the excitatory drive to guide the experience-dependent excitatory network maturation (Chen et al., 2014).

Paradoxically, diazepam treatment during dark-rearing impairs ODP, suggesting that experience or the lack thereof modifies the inhibitory involvement in cortical plasticity (Iwai et al., 2003). Recent studies have demonstrated that prior to the critical period, specific synapses within the cortical network undergo distinct changes. The inhibitory synaptic strength of PV^+^ interneurons onto pyramidal neurons and of somatostatin^+^ (SST^+^) interneurons onto PV^+^ interneurons increases, while the synaptic strength of SST^+^ interneurons onto pyramidal neurons decreases. The excitatory synapses within the local cortical network exhibit a similar decrease in short-term depression in different target cell types (Miao et al., 2016). Additionally, interrogations that affect the maturation of inhibitory systems also lead to changes in expression of particular subtypes of NMDAR subunits. In GAD65 KO mice, the protein level of the NMDAR GluN2A subunit is decreased, whereas diazepam treatment rescues the decreased GluN2A protein levels (Kanold et al., 2009). These findings indicate an intricate interplay between the inhibitory and excitatory synapses within the local cortical circuit during the maturation process that controls the opening of critical periods.

In layer 2/3 pyramidal neurons, the feedforward excitatory and inhibitory drive are equilibrated, resulting in a similar AMPAR EPSC/GABA inhibitory postsynaptic current ratio (Xue et al., 2014). How does the impaired silent synapse maturation in PSD-95 KO mice affect the developmental increase in inhibitory transmission? In PSD-95 KO mice, the maturation of the inhibitory tone (normalized to NMDAR EPSCs) onto layer 2/3 pyramidal neurons during the critical period is similar to that in WT mice (Huang X. et al., 2015). Furthermore, the inhibitory tone in V1 of PSD-95 KO mice increases similar to that in WT mice throughout the critical period. Thus, the prolonged juvenile ODP in PSD-95 KO mice is likely not due to changes in the inhibitory system. ODP in juvenile and adult PSD-95 KO mice is similar to the critical period ODP in WT mice (Fagiolini et al., 2003; Huang X. et al., 2015). Together, these analyses indicate that the increased level of inhibition at the beginning of the critical period is permissive for critical period plasticity (Hensch et al., 1998; Fagiolini and Hensch, 2000) but further increases in inhibition are not instructive and do not end critical periods, because critical period ODP is present lifelong in PSD-95 KO mice, even in the presence of a high “adult” inhibitory tone (Huang X. et al., 2015).

## Critical Period ODP Versus Adult ODP

Since cortical plasticity is also observed in adulthood, it suggests that the refined cortical excitatory networks can further optimize their functional output through progressive training throughout life. At the cellular level, it is known that the rate of synapse turnover is lower in adults, compared to that in critical periods (Holtmaat et al., 2005; Zuo et al., 2005) and the fraction of silent synapses is low, indicating a different molecular and cellular mechanism underlying adult cortical plasticity (Greifzu et al., 2014; Huang X. et al., 2015). It is therefore worthwhile to discuss what cellular and molecular mechanisms govern ODP at different developmental stages.

Prior to the critical period, basic cortical topography has already become organized, and specific visual cortical response properties such as orientation selectivity and ocular dominance are also established, although not yet with their mature features. During the critical period, under physiological conditions, many features of visual function are fine-tuned in an experience-dependent manner, particularly the tuning of neuronal responses to binocular input. It is thought that this tuning is necessary to achieve binocular matching for optimized vision, including binocular vision (Wang et al., 2010; Espinosa and Stryker, 2012).

The initial evidence of the expression mechanism for critical period ODP came from chronic single-unit recordings in kittens (Mioche and Singer, 1989): a short period (6–24 h) of MD led to a decrease of the excitatory responses to the deprived eye, while the excitatory responses to the non-deprived eye remained stable. In rodents, during the critical period, after a short period of MD (1 – 4 days), visual stimulation of the deprived compared to the non-deprived eye results in decreased VEP responses or decreased deprived eye responses, recorded by intrinsic signal optical imaging in the binocular region of V1, contralateral to the deprived eye (Sawtell et al., 2003; Sato and Stryker, 2008). Longer durations of MD (≥7 days), additionally lead to a delayed increase of V1-responses to the non-deprived eye (Frenkel and Bear, 2004; Sato and Stryker, 2008).

After the end of the critical period for ODP, V1 of standard cage-reared mice becomes increasingly less plastic to MD (Lehmann and Löwel, 2008; Sato and Stryker, 2008) and longer MD durations are necessary to induce an ocular dominance shift (Sawtell et al., 2003; Hofer et al., 2006; Lehmann and Löwel, 2008; Sato and Stryker, 2008; Hosang et al., 2018). In clear contrast to the rapid depression of the deprived eye responses in V1 consistently observed in young mice during the critical period for ODP, a hallmark of adult ODP is that MD rather induces potentiation of non-deprived eye responses with no initial depression of the deprived eye responses in V1 (Sawtell et al., 2003; Hofer et al., 2006; Sato and Stryker, 2008). These phenotypic differences between critical period ODP and adult ODP indicate that the underlying mechanisms also differ at different developmental stages (Ranson et al., 2012).

In adults, the mature network and the stabilized synapses do not favor dynamic changes of the excitatory network, spine formation and elimination are at a low rate in layer 5 neuron apical dendrites (Grutzendler et al., 2002; Zuo et al., 2005). Moreover, MD does not induce changes in spine dynamics in layer 2/3 neurons in adult but does so during the critical period (Hofer et al., 2009; Zhou et al., 2017). Additionally, both silent synapses and forms of LTD associated with ODP, which are the two cellular correlates of deprived eye depression during critical period ODP, are diminished in adults (Kirkwood et al., 1997; Heynen et al., 2003; Huang X. et al., 2015; Han et al., 2017). Thus, the silent synapse based refinement of excitatory networks, associated with critical period plasticity, is unlikely to operate in adult standard cage reared rodents.

Depending on the age, MD with different duration leads to non-deprived eye potentiation in adult rodents (Sawtell et al., 2003; Lehmann and Löwel, 2008; Hosang et al., 2018). Different from the critical period ODP, the pre-existing, deprived eye inputs are likely not specifically affected during the initial phase of MD. Additionally, the non-deprived eye potentiation is inhibited by a phosphorylation site mutant of CaMKII T286A, indicating an LTP-like mechanism (Ranson et al., 2012). This is distinct from the non-deprived eye potentiation after prolonged MD (at least 7 days) in juvenile ODP, mediated via homeostatic mechanisms independent of CaMKII activity (Ranson et al., 2012). This homeostatic potentiation is absent in adult ODP. Furthermore, even though hypo-NMDAR function prevented ODP in young adults (Sawtell et al., 2003), the NMDAR signaling in layer 4 principal neurons in adults is dispensable (Fong et al., 2020), suggesting a cell-type specific or layer-specific role of NMDARs in mediating adult ODP. It will require further investigation to identify the underlying synaptic changes and examine whether and how the adult network can recover to the pre-MD condition if MD happens in adulthood, as opposed to the long-lasting deficits caused by MD during the critical period.

A leading hypothesis for the closure of the critical period for ODP so far has been, that a number of so-called “plasticity breaks” (Hensch and Quinlan, 2018), such as the extracellular matrix, Nogo receptor 1 (NgR1), Paired immunoglobulin-like receptor B (PirB), Lynx1, class I major histocompatibility complex (MHC) are expressed and thus prevent critical period ODP (Huh et al., 2000; Pizzorusso et al., 2002; McGee et al., 2005; Syken et al., 2006; Morishita et al., 2010). Loss of function of these proteins leaves the visual cortex in a more plastic state in terms of expressing ODP. However, except for Lynx1 (Bukhari et al., 2015) the mechanisms underlying ODP were not investigated in detail, and it is not yet known whether OD shifts were mediated by deprived eye depression and/or open eye potentiation. Thus, further studies are needed to identify the specific role of the plasticity breaks in critical period ODP. Notably, all these proteins constitute potential candidates to govern the stabilization process of unsilenced gestalt synapses. However, the relationship of these breaks with silent synapses remains unexplored as their role in cortical plasticity was described before the silent synapse-based mechanisms of critical period plasticity were evidenced (Huang X. et al., 2015; Favaro et al., 2018).

Since there is lifelong learning, probably with different expression at network level compared to juvenile plasticity, it remains to be explored how molecular and cellular mechanisms of cortical plasticity differ between critical period and mature networks. Nevertheless, a two-step process emerges: First, during the critical period, the foundation of cortical processing architectures, or the gestalt of the cortical network is established. Second, in adults, the established cortical gestalt enables continuous learning and adaptations of the mature network to ever-changing environments and challenges within the boundary set early on, and by using plasticity mechanisms that are distinct from the principles governing critical period plasticity.

## The Malleability of the V1 Circuit to ODP

Studies using animals raised in diverse environments exhibit different degrees of ODP, implicating malleability in the expression of cortical plasticity. Results from these studies also shed significant light on the distinct expression mechanisms between critical period versus adult cortical plasticity. Most laboratory rodents are born and raised with a small number of animals (up to 5) in so-called standard cages, which are relatively small, usually translucent cages with woodchip bedding, and water and food *ad libitum*. Essentially, these cages represent a rather deprived environment compared to the environment that rodents encounter in the wild, outside the laboratory setting. Enriched environment cages likely provide a more physiological state than the standard laboratory cages. They are bigger, house a larger number of animals, and are usually equipped with running wheels, regularly changed labyrinths and toys to provide a variety of physical, social and cognitive stimulation (Löwel et al., 2017; Stryker and Löwel, 2018).

Deprivation of visual experience is generally used to study the role of experience in cortical plasticity. Either animals are raised in the dark from birth until the experimental time point, termed dark rearing, or animals are put into the dark at various developmental stages and for various durations, termed dark exposure. Combining these manipulations with ODP at different ages, one can query how deprivation of visual input can influence experience-dependent plasticity in young and adult animals.

After around postnatal day 110, mice raised in standard cages are no longer susceptible to MD, even if the MD was prolonged over 20 days (Lehmann and Löwel, 2008; Sato and Stryker, 2008). It is known that giving animals the opportunity of enhanced physical, social, and cognitive stimulation strongly affects physiology, plasticity, and behavior (van Praag et al., 2000; Nithianantharajah and Hannan, 2006; Löwel et al., 2017). Raising mice or rats in an enriched environment alters the expression of several key signaling molecules involved in regulating brain excitability and plasticity (Cancedda et al., 2004), increases the volume of brain areas (Diamond et al., 1964; Beaulieu and Colonnier, 1987), and alters maternal behavior (Sale et al., 2004). Furthermore, enrichment extends ODP into adulthood (Baroncelli et al., 2010a; Greifzu et al., 2014), accelerates experience-dependent V1-activity changes (Kalogeraki et al., 2017), and allows ODP to persist even throughout life (Greifzu et al., 2016). Placing standard cage-reared rodents into an enriched environment as adults, restores ODP (Sale et al., 2007; Baroncelli et al., 2010b, 2012; Scali et al., 2012; Greifzu et al., 2014, 2016), while transferring mice from an enriched environment to standard cages might prevent ODP susceptibility (Kalogeraki et al., 2017).

Some mechanistic insights come from studies on behaviorally interrogated mice with an enriched environment and dark exposure. Both enriched environment and dark exposure, cause a decrease in the inhibitory tone (Huang et al., 2010; Greifzu et al., 2014) and both enable adult ODP. Importantly, with the enriched environment, as little as 4 days of MD in adult mice (>P90) already induced non-deprived eye potentiation (Kalogeraki et al., 2017), and thus accelerated the adult form of ODP, indicating that the decrease of the inhibitory tone facilitates plasticity of the adult cortical network.

**FIGURE 4:**
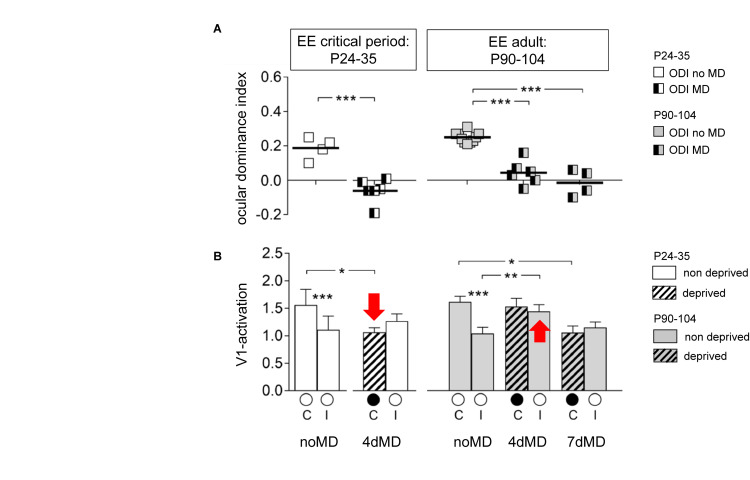
Difference in ODP between juvenile (critical period) and adult mice raised in an enriched environment (EE) (modified from Kalogeraki et al., 2017 use with permission): **(A)** Optically imaged ocular dominance index (ODI). Symbols represent individual ODI-values, means are marked by horizontal lines; values after MD are indicated by half-black squares. **(B)** V1-activation elicited by stimulation of the contralateral or ipsilateral eye without and with MD (black filled circle indicates the deprived eye). In critical period EE-mice, OD-shifts are mediated by a reduction of V1-activation via the deprived (contralateral = C) eye, as in standard cage raised critical period-mice (red arrow pointing down). In contrast, beyond the critical period, OD-shifts in adult EE-mice are mediated via a fast increase of V1-activation through the open (non-deprived, ipsilateral = I) eye (red arrow pointing up). Quantification of V1-activation after stimulation of the ipsilateral (I) or contralateral (C) eye, recorded by intrinsic signal optical imaging in EE-mice of two different age groups before and after MD. Intra- and intergroup comparisons were analyzed either by a two-tailed *t*-test or one-way ANOVA followed by multiple comparisons Bonferroni correction. The levels of significance were set as *p < 0.05; **p < 0.01; ***p < 0.001. Data are represented as means ± SEM.

We would like to stress that ODP of enriched mice after the critical period, is mediated through non-deprived eye potentiation in V1, a hallmark of adult ODP, already after 4 days of MD (Figure 4). Thus, the V1-activity changes in adult enriched mice resemble adult ODP (of standard cage housed-mice) with a faster onset but include an additional not yet characterized component of late depression. After 7 days of MD, reductions of both deprived and non-deprived eye responses in V1 have been observed in adult enriched mice (Kalogeraki et al., 2017). Notably, such reductions in stimulus-induced V1-activity were only seen in mice up to about P300, while beyond P400, only increases in non-deprived eye V1-responses were observed after 7 days of MD (Greifzu et al., 2016). In the earlier work (Greifzu et al., 2014, 2016) ODP was only measured after 7 days of MD, the initial non-deprived eye potentiation was missed, and the results were misinterpreted as providing evidence for “juvenile”-like ODP in adult enriched mice (Greifzu et al., 2014). Given the initial non-deprived eye potentiation after MD in adult enriched mice, the later deprived eye depression is likely mediated by other mechanisms than the juvenile-type of plasticity. Thus, deprived eye input may still exist, though weakened after 7 days of MD. Taken together, ODP in adult enriched mice is different from critical period ODP (Figure 4: open eye potentiation versus deprived eye depression with 4 days of MD), whereas mechanistically similar to ODP in adult standard cage housed-mice (non-deprived eye potentiation), but faster (Kalogeraki et al., 2017).

Consistent with previous work in cats (Cynader and Mitchell, 1980; Timney et al., 1980; Cynader, 1983) and rats (Fagiolini et al., 1994), dark-reared mice display a delayed critical period (Chen et al., 2014), and potentially the window for ODP is prolonged into “adult” age (Fagiolini et al., 2003; Gianfranceschi et al., 2003; Favaro et al., 2018). Furthermore, when a period of dark exposure is started in adulthood, MD can again induce ODP in adult standard cage housed rodents (Guire et al., 1999; He et al., 2006; Stodieck et al., 2014). Remarkably, dark exposure after P70 leads to functional improvements in rats with amblyopia (He et al., 2007). After 3 days of MD, deprived eye responses are reduced as well as non-deprived eye responses increased (He et al., 2006). In mice, after dark exposure, ODP is mediated by an increase of non-deprived eye responses in V1 after 7 days of MD (Stodieck et al., 2014). The simultaneous expression of both, reduction of deprived eye responses and increase of non-deprived eye responses after short MD in dark exposed adults, is distinct from both critical period and adult ODP with an enriched environment.

Together, these results show that both enriched environment and dark exposure (deprivation of visual experience) enhance ODP compared to standard cage rodents. It is intriguing how the two seemingly opposite manipulations lead to a phenotypically similar outcome. While mechanisms, such as metaplasticity with experience-dependent modifications in NMDAR subunit composition by dark rearing (Philpot et al., 2001; Corlew et al., 2007), and changes in inhibitory tone with the enriched environment (Greifzu et al., 2014) have been proposed, further experiments will be necessary to elaborate the mechanistic differences and potential similarities in the plasticity after dark exposure and that in an enriched environment. Notably, no induction of silent gestalt synapses was observed with enriched environment exposure (Greifzu et al., 2014) indicating that at least environmental enrichment is not effective in rejuvenating the cortical network for critical period plasticity.

## Critical Periods Configure Memory Traces, Shaping Adult Perception Capabilities

The process of critical period network refinement through the step-wise maturation of excitatory synapses is essential for proper perception in adulthood. For example, juvenile barn owls can learn abnormal associations between auditory cues and locations in visual space if their visual input is displaced by wearing prism goggles, and these abnormal associations are recalled in adulthood if the owls re-experience the displaced sensory inputs by again wearing prism goggles (Linkenhoker et al., 2005). However, this adaptation is hampered if the owls experience the abnormal association for the first time in adulthood when plasticity is limited. Thus experience during critical periods shapes the gestalt of neural networks which is essential for the ability to perceive certain sensory associations later on.

In the rodent visual system, visual perception, as measured by orientation discrimination, is impaired if critical periods close precociously as in PSD-93 KO mice, or if the closure is impaired as in PSD-95 KO mice (Huang X. et al., 2015; Favaro et al., 2018). In the absence of both PSD-93 and PSD-95, visual perception in mice is further compromised and only innate visual capabilities remain, indicating that either PSD-93 or PSD-95 is required for establishing gestalt synapses (Favaro et al., 2018). Further studies will be needed to explore the features of synapses lacking both PSD-93 and PSD-95, and whether only innate synapses are left in double KO mice. Furthermore, mice with a postnatal knock-down of neurogranin in the visual cortex were impaired in perceiving a visual cliff (Han et al., 2017). While it is not yet clear whether the loss of neurogranin specifically affects pruning of gestalt synapses, the impaired silent gestalt synapse maturation is consistent with their role in visual perception.

Binocular matching of orientation tuning and binocular vision require experience-dependent cortical plasticity during critical periods and if this is prevented by dark rearing or genetically impairing critical period refinement, these visual features are otherwise impaired in adulthood (Kaye et al., 1981; Mower et al., 1985; Crair et al., 1998; Wang et al., 2010; Han et al., 2017). However, maturation of visual acuity, a visual function thought to be controlled at the thalamic level, appears less sensitive to either genetic or sensory interrogations of the time course of critical periods, as it is unaffected by dark-rearing, or loss of either PSD-93 or PSD-95 (Rochefort et al., 2011; Kang et al., 2013; Huang X. et al., 2015; Favaro et al., 2018). While more detailed mechanistic studies are still needed, these results highlight the critical function of the signaling scaffolds PSD-93 and PSD-95, as well as neurogranin in coordinating the proper pace and balance of silent-synapse based cortical excitatory network maturation during critical periods (Huang X. et al., 2015; Favaro et al., 2018; Han et al., 2017). The specific impairments in this process set a premise to untangle the cellular mechanisms of critical period principal neuron network refinement.

Judging from the clinical experience in treating neurodevelopmental disorders, merely inducing or enhancing adult plasticity is unlikely sufficient for long-term modification of the network, as a potential pathway to correct network dysfunction caused by developmental deficits. Thus, one provocative idea is to try to rejuvenate networks by engaging critical period plasticity to reorganize/repair connectivity in adulthood. Hijacking the rejuvenation mechanism promises a potential approach that could lead to long-lasting reorganization of the cortical network to correct network dysfunction, such as amblyopia but also other neurodevelopmental disorders that are caused by developmental deficits. Knocking down PSD-95 in adult mice was already shown to restore the fraction of silent synapses to the eye-opening level, and also to reinstate critical period ODP (Huang X. et al., 2015). Together, these observations suggest that the network can be reverted to express critical period plasticity. Additionally, a rejuvenation of the excitatory network has also been successfully induced in the brain’s reward circuit under pathological conditions with drugs of abuse. Cocaine challenge induces newly formed silent synapses in this circuit, which leads to long-lasting network modifications that contribute to the obsessive drug-seeking behavior in a rodent model of addiction (Huang et al., 2009; Huang Y. H. et al., 2015; Wright et al., 2020). Both approaches have disadvantages due to the lack of perceptual maturation when PSD-95 levels are low, and the potential of drug addiction. Nevertheless, understanding the molecular underpinnings and cellular processes that guide excitatory network maturation during critical periods will help to figure out whether adult neural networks can be rejuvenated for network reorganization/repair.

## Conclusion

Using the critical period for ODP in the primary visual cortex as a model system, the field has gained significant insight into understanding the experience-dependent refinement of cortical networks during postnatal development. This process builds the fundamental architecture for achieving optimal functional output after later progressive training (Figure 1). Both excitatory and inhibitory systems operate hand in hand to open the critical period, and to define the duration of critical period synaptic plasticity. In recent years, it also became clear that the plasticity mechanisms during the critical period are different from those in adults. We propose a model with two distinct types of synapses, *innate synapses* that establish rudimentary networks with innate function, and *gestalt synapses* that govern the experience-dependent refinement process. The maturation of silent gestalt synapses, transitioning via experience-dependent unsilencing and stabilization is a fundamental step in strengthening functional ensembles of the cortical network. The generation, maturation, or pruning of gestalt synapses governs experience-dependent network maturation, and is the essential mechanism for the developmental refinement of cortical principal neuron networks (Figures 2, 3). While this review focuses on the functional analyses of silent synapses in cortical layer 2/3 pyramidal neurons, silent synapses are prevalent at different developmental stages also in cortical layer 5 (Anastasiades and Butt, 2012), hippocampus (Isaac et al., 1995; Liao et al., 1995), and nucleus accumbens (Huang et al., 2009). Experience-dependent spine dynamics, as proxies of the plasticity of excitatory networks, have been observed in both cortical layer 2/3 and layer 5 (Holtmaat et al., 2005; Zuo et al., 2005). Potential differences in network dynamics in different layers and brain regions warrant further investigations. This silent synapse-based network refinement is likely generalizable to all excitatory networks in sensory cortices, as well as to higher-order cortical networks controlling cognition and executive functions, such as the prefrontal cortex. Despite extensive studies on critical period plasticity and the role of silent synapses in Hebbian plasticity, it is only recently that their defining role in critical period plasticity was revealed, opening the door for further studies to elucidate the underlying molecular mechanisms.

While our new working model raises many questions and challenges, it also provides testable hypotheses and may thus guide further research. First, although we deduced different functional categories of synapses and transitions through different states during experience-dependent network maturation, it remains unknown how the different synapse categories and states are defined at the molecular level. While lack of PSD-95 prevents the transition from unsilenced gestalt synapses to stable gestalt synapses, little is known about the key signaling cascades that control this transition. Our model postulates that unsilenced gestalt synapses are the substrates for LTD and pruning during critical period refinement, which in turn constitutes the expression mechanism of deprived eye depression during MD (Figures 2, 3). Based on this model, the unsilencing and stabilization of gestalt synapses are distinct processes. While LTP drives AMPARs into the gestalt synapses, the stabilization process goes beyond the LTP time window to finalize the maturation.

Our recent studies provide some initial insights into the molecular mechanisms governing experience-dependent maturation of excitatory synapses, and identified three key players: neurogranin, PSD-95, and PSD-93 (Figure 2B). While PSD-95 drives the maturation of silent synapses, PSD-93 opposes this function. Neurogranin is a key switch governing the fate of gestalt synapses for maturation or through regulating post-synaptic calcium/CaM-dependent signaling. Further investigations of the molecular landscape associated with these key players will help to further identify molecular mechanisms for the different state transitions during the experience-dependent synapse and network maturation process, such as synapse pruning and synaptogenesis of gestalt synapses during critical periods and how they interact with synapse maturation.

New players are emerging from recent studies, yet, it remains critical to apply the molecular approaches combined with functional analyses to further elucidate how the identified targets regulate excitatory network maturation and perceptual capabilities. Furthermore, cellular and molecular identities involved in glia and astrocyte signaling in regulating postnatal synaptogenesis and synapse elimination warrant further investigation. Finally, the intriguing array of proteins that put a break to critical period plasticity should be investigated in light of synaptic state transitions. Together, the new analyses may answer the key question whether it is possible to preserve or reactivate the generation of silent synapses in the adult brain, to rejuvenate the network gestalt for a critical period-like plasticity to “repair” abnormal configurations of the neuronal networks.

## Author Contributions

All authors listed have made a substantial, direct and intellectual contribution to the work, and approved it for publication.

## Conflict of Interest

The authors declare that the research was conducted in the absence of any commercial or financial relationships that could be construed as a potential conflict of interest.
